# Advancing residents’ use of shared spaces in Nordic superblocks with intelligent technologies

**DOI:** 10.1007/s00146-022-01604-x

**Published:** 2022-12-02

**Authors:** Jouko Makkonen, Rita Latikka, Laura Kaukonen, Markus Laine, Kaisa Väänänen

**Affiliations:** 1 Unit of Computing Sciences, Tampere University, Tampere, Finland; 2 Faculty of Social Sciences, Tampere University, Tampere, Finland; 3 Faculty of Management and Business (MAB), Tampere University, Tampere, Finland

**Keywords:** Nordic superblock, Shared spaces, Human-Centered Artificial Intelligence (HCAI), Intelligent technologies, Neighborhood, Smart cities, Residents

## Abstract

To support the sustainability of future cities, residents’ living spaces need to be built and used efficiently, while supporting residents’ communal wellbeing. Nordic superblock is a new planning, housing, and living concept in which residents of a neighborhood—a combination of city blocks—share yards, common spaces and utilities. Sharing living spaces is an essential element of this approach. In this study, our goal was to study the ways in which intelligent technology solutions—such as proactive, data-driven Artificial Intelligence (AI) applications—could support and even motivate the use of common areas in superblocks. To this end, we conducted a two-phase qualitative study: in the first phase, potential superblock residents (*N* = 12) shared their perspectives of sharing of living spaces in general, and more specifically of how intelligent technologies could support sharing spaces. In the second phase, two workshops with experts (*N* = 7) were held to gather understanding of possibilities of intelligent technologies in meeting the residents’ expectations of space sharing. The results illustrate space sharing and communality as supportive factors for one another, enabled but also complicated by social interaction. Major possibilities for intelligent technologies to advance space sharing were seen in organizing the use of spaces and facilitating social interaction in the community. As an outcome, four roles incorporating several use purposes of intelligent technologies were found. The findings can inform the Human-Centered AI (HCAI) research and design improving sustainable living in future urban neighborhoods.

## Introduction

The global growth of urban population increases the need for buildings occupying people (Northfield 2016) and effective use of living spaces (McDonald 2008). At the same time, there is an increasing trend of living alone (e.g., Official Statistics of Finland 2020), and individualistic way of life has reduced interaction between people. Further, in the coming years, governments will need to tackle climate change (Compston and Bailey 2013), and accelerating inequality in cities (Glaeser et al. 2009). Addressing these phenomena requires innovations in urban development and in the ways in which residents use the city. Intelligent technologies are scarcely applied to this area and their possibilities should be explored and potential investigated. By intelligent technologies, we refer to a range of proactive, data-driven technology-based applications that involve some forms of Artificial Intelligence (AI) in their functionality.

On a larger urban planning scale, scholars have proposed to focus on designing and building of resident-friendly neighborhoods, where sharing of spaces for certain live activities can be supported. Indeed, sharing of living spaces could improve both social and environmental sustainability through more effective use of living spaces and by increasing interaction between people. Famous examples of this are Barcelona superblocks, where the idea is to create car-free public space inside nine city blocks in the existing urban structure (see for example Rueda 2019). The concept of *Nordic superblock* refers to a new planning, housing, and living concept developed in Hiedanranta, which is an innovation- and sustainability-oriented, rapidly developing city area in Tampere, Finland. It is a combination of mixed-use residential blocks that share yards, common spaces, services, and utilities (Sjöblom et al. 2021). In the Nordic superblock development, the idea is to create new urban structure, and to bring a novel scale of co-operation into urban planning and development: number of blocks would now share the resources previously shared between residents of one building. Instead of building the usual community room for each building, the money reserved for it could be used for realizing a variety of shared spaces around the block, like a shared pool, sauna, workshop, neighborhood café, and gym (Alatalo et al. 2018).

Previous studies have less focused on sharing of living spaces, although discussion around collaborative housing or co-housing has been quite lively (Lang et al. 2020). It has focused on analyzing the community and the process, but not so much on the features of the shared spaces that are generated in collaborative housing projects. Therefore, more knowledge is still needed on the preferred ways of using shared spaces. Another still scarcely investigated area is the role of intelligent technologies in shared spaces in residential buildings. AI-driven applications are already in use in many smart housing solutions such as adaptive and data-driven heating and lighting automation (Moreno et al. 2014). Intelligent technologies hold great yet unexplored potential in shared spaces in residential settings, also in supporting and even enticing the use of common areas in superblocks.

The goal of this qualitative study was to create insights to fill in the detected research gaps by investigating the residents’ expectations towards shared spaces, and the potential role of intelligent technologies to support space sharing. This research belongs to the recently growing area of Human-Centered AI (HCAI) in which human values, needs and the wider sociocultural context is taken into the focus starting from the early phases of AI solution development (Schmidt et al. 2021; Xu 2019). The specific context of this research is the *Nordic superblock*, described above. In this study, our focus is on shared spaces as common spaces and areas on the scale of residential neighborhood. In addition, our definition includes sharing living and social spaces, as presented in Chan and Zhang’s (2021) vectors of the socio-spatial dimensions of sharing.

The study was conducted in two phases. The aim of the first phase was to investigate the needs, expectations, and complications towards shared spaces and use of intelligent technologies in space sharing. The aim of the second phase was to assess the design implications for AI-based intelligent technology solutions for advancing the use of shared spaces. In line with these aims, we set the following research questions:

- RQ1: What expectations residents have towards space sharing in their residential area?
- RQ2: What are the residents’ perceptions of using intelligent technologies that could advance the use of shared spaces?
- RQ3: Based on the findings of RQ1 and RQ2, what are the design implications for intelligent technology solutions for advancing the use of shared spaces?

Studying residents’ expectations towards shared spaces (RQ1) aims at understanding the context and user needs of the intelligent technologies that could advance the use of shared spaces whereas RQ2 focuses on the perceptions of using these intelligent technologies based on residents’ expectations. Based on the use context and used needs as well as expectations and perceptions, RQ3 draws conclusions for designing such intelligent technologies in the form of design implications. This study contributes to the field of Human-Centered AI by increasing the understanding of the forms in which AI-driven intelligent technologies can fulfill user needs for social interaction in shared residential spaces in the urban context.

## Background and related work

The first section of this chapter describes the concept of shared spaces in the context of this study, Hiedanranta area in the City of Tampere, and the related research on housing schemes and resident participation. The second section presents findings of previous urban space studies on how social interaction can be supported in shared spaces. The third section presents how AI-driven solutions can be used to benefit urban living, and more specifically to advance sustainability and sociability in a smart city.

### Shared spaces within housing schemes of Hiedanranta superblocks

**Fig. 1:**
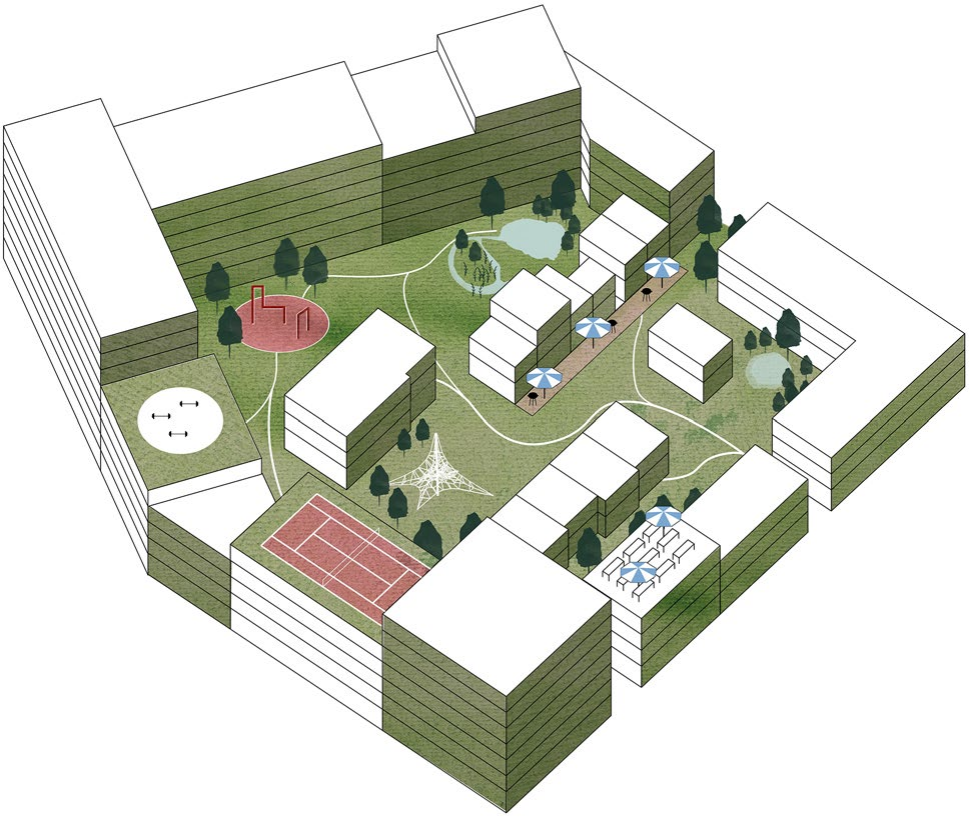
An early vision on sharing spaces between several houses in a Hiedanranta superblock in Tampere. (Source: Hiedanranta Master Plan 2020. Copyright: Tampereen kaupunki 2020)

Hiedanranta is a former industrial area, bought by the City of Tampere in 2014, to be developed into a new city district for around 21,000 residents and 8000 workplaces (City of Tampere 2020). The city’s vision is to turn Hiedanranta into a sustainable and smart urban area, that will function as a Western city center of Tampere. Future residents will be living in superblocks spanning more than one ordinary city block, which will according to the Hiedanranta Master Plan (2020) support sustainability and collaborative decision making. Superblocks are supposed to share communal spaces such as saunas, working spaces, laundry, common kitchens, hobby hub; mobility services such as ebikes, shared cars; and tool and gear libraries. Building of the first residential buildings is planned to begin in year 2023. Figure 1 depicts initial sketches of a Hiedanranta superblock.

Resident participation has been rarely employed in early stages of planning in the Finnish history of housing (Kuoppa et al. 2020). In the past, developing suburbs unattached from the central urban structure has been characteristic for urban development. This has led to criticism of not bringing people together socially, and a need for more compact and connected city structure (Laine et al. 2020; Saarivirta et al. 2021).

In contrast to the background of the history of Finnish urban development, the case of Hiedanranta is unique. The idea of the Nordic superblock as a model for housing in the area was born out of resident participation. In the planning process of Hiedanranta, expert-resident workshops were organized as a form of resident participation. In these workshops the idea of utilizing superblocks as a housing model in Hiedanranta was originated and improved upon. This happened at the early stages of developing the area, which was rare, because it happened before the master plan for Hiedanranta was composed. Usually, residents have opportunity to comment urban development only after an almost ready draft plan has been and later, in a form of a complaint (Sjöblom et al. 2021).

As a continuity to the research on the Nordic superblock, our research sheds light on the little researched topics of residents’ preferences for shared spaces in housing, and technological solutions that could support them. Resident’s preferences have been examined from the viewpoints of their personal accommodation and its features (Kuoppa et al. 2020) and collaborative housing (Laine et al. 2020). Resident participation has already been proven beneficial to urban development in the context of the Nordic superblock (Sjöblom et al. 2021), but resident’s preferences for shared spaces in the context of housing have not been investigated before.

### Shared spaces and social interaction

The term shared space is used broadly in the literature, referring to for instance public green areas (Holder and McGillivray 2019), roads and traffic arrangements shared by motorized and non-motorized forms of traffic (Luca et al. 2012), shared social spaces (e.g., co-working spaces; Chan and Zhang 2021), and communal or common areas and spaces in residential areas (Rabinowitz 2012). The focus of this study is on the latter two. Chan and Zhang (2021) present socio-spatial dimensions of space sharing on three spatial scales: urban sharing with practices that are city wide; sharing a living space in domestic scale; and shared social spaces that are defined by advancing and resulting to social sharing. The scale of residential neighborhood in our study connects to sharing living spaces and social spaces.

Nugent (2012) addresses the issue of residents not using the shared spaces by investigating the characteristics of successful shared spaces in the context of student campuses. Found attributes were proximity, visibility and openness of the space, including multiple activities in one space, optimal size of the space, equal distribution and variety of spaces, experience of ownership of the space, pleasant quality of the space, and comfortable, flexible and appropriate furniture of the space.

Previous research has found connections between shared spaces and increased social wellbeing. Stupar et al.’s (2020) findings reveal that placemaking process can activate the use of shared spaces and, because of that process, they have potential to increase interaction and co-operation as well as strengthen community and sense of belonging. Further solutions in built environment can have an impact on social wellbeing. Vegetation and green areas in shared spaces increases the use of those shared spaces and formation of social contacts between neighbors (Kuo 1998). Walkability on the streets and in shared residential areas has been found to increase social interaction, and mobility in general is important in reducing loneliness, especially in the case older people (Curl et al. 2015; van den Berg et al. 2016). Makerspace and hackerspace types of social workspaces, by definition, intend and have potential to increase social interaction and co-operation (Chan and Zhang 2021; Seo-Zindy and Heeks 2017) and they are also examples of shared spaces that connect the use of advanced technologies and activities.

### Human-Centered AI services for sustainability and sociability in the smart city

The concept of smart city has been explored widely on the global scale. The goal of smart cities is to offer their citizens improved quality of urban life (Bakici et al. 2013) and sustainability in the city operations by optimizing the multitude of city processes and operations (Andreani et al. 2019). By involving their citizens, smart cities can act as platforms to generate new ideas for products and services based on open data and intelligent technologies, as well as develop and test them in living labs involving citizens in the co-creation process (Bakici et al. 2013).

In many smart city visions, advanced digital technologies are expected to broadly solve urban sustainability problems. Such visions have been critiqued in the urban studies literature for being techno-optimistic, but Martin et al. (2018) found in their literature review that the potential to empower and include citizens is the key to implement forms of smart and sustainable urban development that emphasize environmental and social equity.

The smartness in the smart city arises from the use of AI-driven digital technologies to implement the co-created smart city solutions. The prominent intelligent technologies are Machine Learning (ML) algorithms, embedded sensors, Internet of Things, as well as associated data security and connectivity solutions (Gharaibeh et al. 2017; Ahad et al. 2020). Citizens can interact with the services based on their mobile devices, public displays, and intelligent user interfaces embedded in the urban space. AI-driven solutions aim at supporting people’s wellbeing, fluency of their everyday lives (Berryhill et al. 2019), city sustainability through resource optimization, inclusivity, e.g., through smart mobility (Benevolo et al. 2016), and sustainable use of buildings (Radziejowska and Sobotka 2021).

To make the services acceptable to end-users, they need to be developed along the principles of HCAI (Schmidt et al. 2021). One of the key principles in this approach is to maintain citizens’ autonomy when using AI (Väänänen et al. 2021), including keeping the control of the user–system interaction in the hands of people (Shneiderman 2020). Involving people in the early stages of AI service development is another central principle of HCAI, and hence studies of end-user needs are needed. Lehtiö et al. (2021) found in their study of citizen perceptions of AI in the Smart City context that people do not like AI or people behind it monitoring them. Furthermore, citizens to not want AI to mimic people, and that they will avoid using AI if they consider the risk too high.

Relating to the specific understanding of the potential of the AI-driven solutions in the contexts of shared spaces and sociability, *urban informatics* studies the intersection of place, technology, and people (Foth et al. 2011). Johnstone et al. (2016) found that in community-driven creative hubs, the connection of technology and people has been manifested by communication technologies and social media (Johnstone et al. 2016). Fatah gen Schieck et al. (2011) have pointed out that making digital identities of users of a shared space visible to other space users increases social interaction but also concerns about privacy. In some cases, digitally supported community interaction has been experienced intrusive and forced (Johnstone et al. 2016). Furthermore, data gathering in public spaces has been found to cause anxiety, due to experienced control of residents in smart cities (Mann et al. 2020).

Citizens appreciate services that aim to maintain sense of safety and stability in their social environment and make their everyday life easier (Ji et al. 2021). Promising services promote communal bond or activity, support citizens’ personal interests and wellbeing through access to learning resources as well as smart, high-quality healthcare, and encourage innovative development in urban environments (Lytras et al. 2019). Acceptance of smart city services requires that citizens are assured that their privacy is guaranteed and that the cost of these services does not outweigh their benefits (Habib et al. 2020). Furthermore, smart services need to adjust their proactivity level according to the character of the application area, for example when dealing with sensitive data (Meurisch et al. 2020).

Technology-mediated social interaction between co-located people has been addressed by the research of *social technologies* (Olsson et al. 2020). Such context-aware technologies have been investigated in various contexts of use, such as matchmaking in leisure activities (Paasovaara et al. 2018) and forming effective work teams (Koivunen et al. 2021). Algorithmic, Machine Learning, and sensor-based solutions can be used to advance sociability is shared situations, but people’s privacy concerns must be carefully taken care of.

The research gap addressed by this research is in the lack of understanding of user needs for social interaction in shared residential spaces, and previously unexplored possibilities of technology-mediated, AI-driven solutions in this context.

## Study design

The goal of the study was to create novel understanding of the possibilities of intelligent technologies to support the use of shared spaces in neighborhoods. By following the research through design approach (Zimmerman and Forlizzi 2014) we explored and formed novel, design-relevant insights of potential intelligent technology concepts based on empirical research with potential end-users and experts. Qualitative research methods were chosen to gain in-depth understanding of the phenomena related to residential living and potential of intelligent technologies.

Semi-structured interviews were selected for an early-stage evaluation of user’s expectations of intelligent technology solutions in a new use context. Interviews aimed at exploring potential areas of use and probing the attitudes towards intelligent technologies in the sphere of residential sharing. Co-design workshops with experts were chosen as a method to form design implications from the findings of the interview study.

### Interview study

In the first phase, 12 semi-structured interviews were conducted among potential future residents of Hiedanranta area. The interviews addressed the participants’ needs, expectations, and complications towards shared spaces and use of technologies in space sharing (RQ1 and RQ2). Recruitment channels used in the study included social media, email lists, and personal contacting. Participant recruitment targeted people from residential areas or buildings that resembled the planned Hiedanranta neighborhood. Most of the interviews were conducted by telephone, and a few interviews also utilized a video connection. All interviews lasted approximately an hour, were recorded and transcribed.

Of all participants, 7 were females and 5 were males, and their age ranged from 24 to 80+ years old. The majority (*n* = 8) of participants lived in Tampere while some came from the capital Helsinki (*n* = 4). Most of the participants (*n* = 8) lived with someone (e.g., a partner, a flat mate or children), and a few lived alone (*n* = 4). The housing type of the participants included apartment buildings, terraced houses, and detached houses. Most participants lived in an owned (*n* = 8) and some in a rental (*n* = 4) place of residence.

The method of analysis was thematic analysis (Braun and Clarke 2006; Gavin 2008) which was considered best to serve our research objectives. Following the analysis phases provided by Braun and Clarke (2006), we began the analysis with familiarizing ourselves with the data. Second, the first three authors, representing three different disciplines, independently generated initial codes for analysis into Excel. Third, the first author searched for and created broader themes. Fourth, three first authors again reviewed the final themes. Hence, the analysis process formed a multidisciplinary triangulation of researchers.

### Co-design workshops

In the second phase, the key findings of the interviews on user’s expectations, needs, and use context were brough to two co-design workshops that were conducted among experts, namely user experience (UX) and human–computer interaction (HCI) researchers with experience in artificial intelligence. The aim of the workshops was to assess the design implications for AI and intelligent technology solutions for advancing the use of shared spaces (RQ3). In practice, a canvas in Mural-platform was used in the remote workshops, where the experts were presented the main findings of the interview study, i.e., residents’ expectations, needs or wishes, and complications towards space sharing and technologies in residential context, expected use purposes of intelligent technologies, and the raised social issues regarding sharing in residential context. With the help of the canvas, it was examined how challenges that arose in the interviews could be solved by using AI-driven intelligent technologies and taking the needs and wishes of residents or other relevant findings into consideration. The challenges were *enhancing space sharing or use of shared spaces*, *advancing people meeting each other*, *advancing activities, meeting while doing, and use of adaptable multipurpose space,* and *advancing space sharing among the whole neighborhood*. With this approach, the goal was to base the design implications on both residents’ and experts’ perspectives. Experts were recruited via personal email invitations. The rationale for involving experts with experience in AI was to find future directions and roles for intelligent technologies in particular, instead of current and commonly known technologies, which was expected to require more advanced knowledge on intelligent technologies.

The two co-design workshops involved seven participants in total, four in the first one and three in the second one. The sample consisted of researchers at different stages of their research career from PhD students to senior researchers. The first workshop was conducted in English and the second in Finnish language. Both workshops were performed online, lasted 1 h and 30 min, were recorded and transcribed. The first author of the paper facilitated both workshops.

Qualitative approach was again used for data analysis. The aim of was to form the main design implications for AI-technology and intelligent technology solutions for advancing the use of shared spaces. First, the three authors reviewed, clustered, and categorized the workshop material and independently generated initial drafts for design implications. Second, the suggested design implications were discussed within the research group, merged, and fine-tuned. Workshop findings were again evaluated in the light of interview findings, seeking for similarities and conflicts. Potential purposes, design implications and roles of technologies were concluded based on both workshop and interview findings.

## Findings

Findings reveal a connection between community of residents, social interaction, and functionalities of the spaces as major influencers on space sharing. Participants saw possibilities for technologies to support space sharing especially through supporting communality, facilitating the use of shared spaces and activities in them, and facilitating and supporting social interaction. As design implications roles of a community sheriff, a matchmaker, a facilitator, and a tutor were found for intelligent technologies based on both interviews and co-design workshop results.

The findings are presented under each research question. Residents’ expectations towards space sharing in their residential area (RQ1) form a context and develop to needs considering the intelligent technologies that could advance the use of shared spaces (RQ2). Design implications (RQ3) take into consideration the expectations and perceptions towards both, shared spaces, and intelligent technologies in the context. Findings of RQ1 and RQ2 are based on the interview study and findings of RQ3 both interview study and co-design workshop.

### Residents’ expectations towards space sharing in their residential area (RQ1)

Participants’ expectations towards space sharing targeted the spaces, functions, and purposes of spaces and other users of spaces. Participants were in general open towards having and using shared spaces in their neighborhood. Participants expect shared spaces in their residential areas to work as extensions of home that have functions that meet their needs. They prefer meeting other people in shared spaces during activities. Space sharing was expected to support communality and communal space sharing. Social interaction was considered supportive and complicating factor. Benefits in the form of efficiency and personal impacts were expected.

In the following, three main themes related to residents’ expectations towards space sharing (RQ1) are presented: “Extra space, functions and meeting people while carrying out activities attract people to use shared spaces”; “Space sharing challenges the community of residents to develop in social interaction and communality”; and “Personal benefits and obstacles of space sharing.”

#### Extra space, functions and meeting people while carrying out activities attract people to use shared spaces

Participants considered shared spaces as *functional extensions of home* that enable *activities*.

[shared spaces mean that] people have in their direct living environment, well, home extensions, and huge number of possibilities compared to typical homes of people. (P1)

Need for extensions of home derives from experienced limitations of own home, which participants experienced as a major motivation for using shared spaces. The limitations can relate to utilities (e.g., doing laundry) or hedonic factors (e.g., leisure, entertainment). Home might not provide enough size, suitable layout, wanted *functionality* (e.g., sauna, exercise) or the functionality can be improved when implemented in shared spaces (e.g., size of a sauna, large and professional level kitchen), when the costs are shared. Multiple participants mentioned their willingness or need to do “messy” or “dirty” chores or activities, for instance, bicycle maintenance or woodcraft; however, there is not suitable space for them in their home. Shared spaces are also expected to provide *stimulus*, for instance, entertainment, view or connection to nature, that home does not.

The needs towards shared spaces were similar than found limitations, added with social dimension. Participants described *utility*, *leisure,* and *social needs* towards spaces. All participants expressed willingness to fill their social needs by *meeting people in shared spaces*. For many, meeting other people was one of the most important or the most important reason for using shared spaces in living areas or even for the spaces to exist. Using shared spaces was seen as a possibility for meeting both new people and already familiar people. Some participants mentioned explicitly, that getting to know neighbors is an important thing not just personally but also for the communality in the neighborhood.

However, meeting others was experienced more natural and fluent when it is connected to doing something instead of meeting per se. Most participants, preferred *meeting others while doing activities*, for instance sports, when the activity functions as a common factor, facilitates the social interaction.

Like in general in hobbies, one might get to know others so that there is the common factor of doing something that they like. And not just like ‘Lets socialize!’, and that might become awkward and artificial. (P4)

**Fig. 2:**
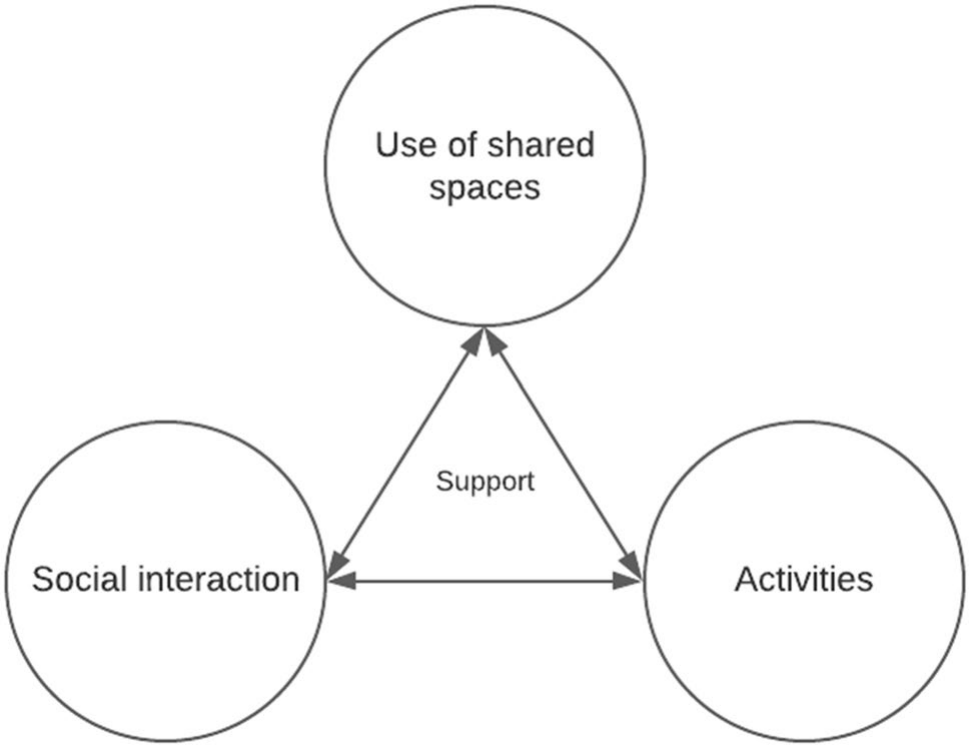
Activities and social interaction were motivations for using shared spaces. Activities in shared spaces were considered to catalyze social interaction. Participants experienced that those support each other

Overall, participants connected shared spaces tightly to their *functions* or the *activities* that can be done in those. Activities in spaces were desired. Activities and functionalities in shared spaces serve also purpose of *catalyzing social interaction*. Participants considered that events around some activity like game nights or barbeques help neighbors to get to know each other and advance communality in the building as well as activates the use of spaces. These interconnections of the use of shared spaces, social interaction and activities are illustrated in Fig. 2.

Participants associated various activities to meeting others in shared spaces: for instance, different hobbies including sport and exercise, handcraft and music or gardening, entertainment or stimulus activities like game nights or watching sport, utilities like bike maintenance or communal work in the yard or barbeque. Further, learning together or from others, namely, sharing knowhow or having peer support, were expected as additional benefits of meeting others while doing activities. Learning needs were associated with, for instance, doing handcraft or using technology.

Interviews revealed also more detailed expectations towards space sharing in residential areas. Participants expected resident involvement in developing the activities in shared spaces or even in planning the space, if possible. Flexible, adaptable, and multipurpose spaces were seen as a solution for meeting versatile needs. As requirements towards shared spaces participants considered that spaces need to be easy to go to and otherwise feasible, and they were expected to be sustainable, durable, and efficient. Space sharing was expected and experienced to save space, money, resources, and energy.

I would assume that in our house people have accepted smaller apartments because they know that there are shared spaces they can use. (P1)

Feasibility includes physical and mental aspects; if the space is far from resident’s home or otherwise difficult to go to, or if it is unpleasant, it might become an obstacle of use. Participants expected enough capacity from spaces, or a possibility to see the space usage rate real time, and easy reservation when applicable. This was related to fluent use of spaces, but also to avoidance of excessive contacts due to the COVID-19 pandemic that was ongoing during the interviews.

#### Space sharing challenges the community of residents to develop in social interaction and communality

**Fig. 3:**
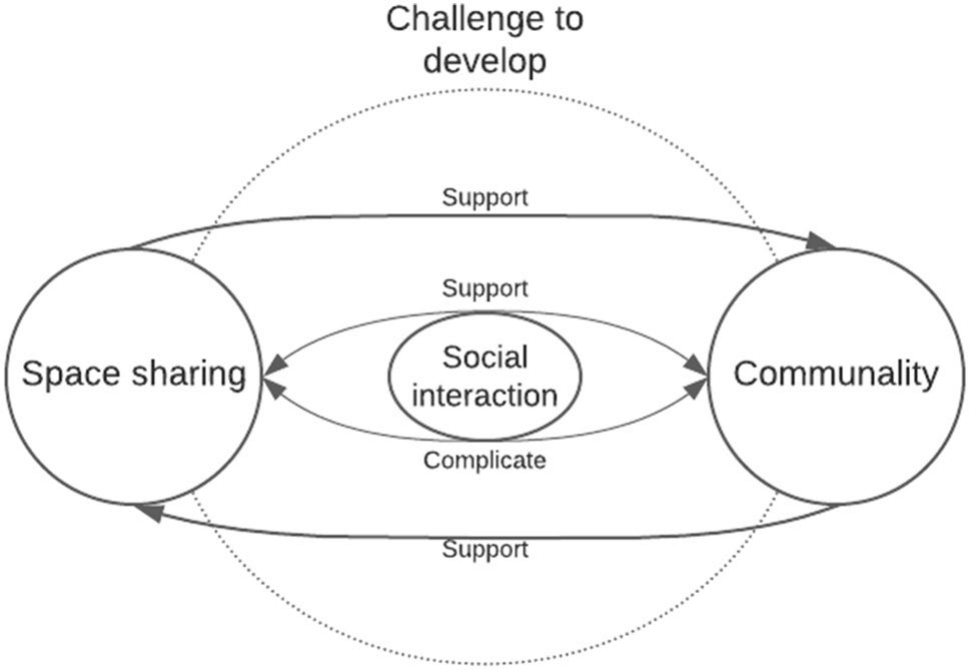
Space sharing is expected to support communality and communality space sharing. Social interaction both supports and complicates this relation. These interrelations challenge the residents to develop their social interaction and communality

Participants expect that social issues in a community of residents’ impact space sharing by fostering the communality, but also complicating it—and vice versa: space sharing can influence community and support communality. Space sharing challenges the community of residents to develop in social and mental maturity and residents to take responsibility, to space sharing to be successful. Social interaction is both supporting and complicating factor for space sharing. Figure 3 depicts this interrelation between space sharing, communality, and social interaction.

*Enhancing communality was emphasized as a major expected benefit of shared spaces* among interviewees. As part of it, participants referred to opportunities to learn to know their neighbors in person, which could result in forming new social relations with neighbors.

At least perhaps the rise of community spirit, or communality. You might get to know [people in] the neighborhood, and that might result in finding friends and getting help from neighbors. (P4)

Participants expected that shared spaces could increase social interaction between residents. Further, it was discussed how shared spaces could narrow the gaps and increase understanding and tolerance between people of different ages, from different ethnical or cultural backgrounds or with different lifestyles and habits. Moreover, some participants considered that shared spaces could, following the potential growth of communality, create and enhance local self-expression. By this, they meant self-planned and made activities, culture and influencing local matters. Participants considered enhanced communality important for all in the neighborhood and highly important for elders, people who are lonely and people with special needs. Participants connected enhanced communality to wellbeing, mainly social, but also physical and mental wellbeing.

*Communality was also expected to support space sharing in the neighborhood.* A few participants mentioned that nurturing communality actively advances space sharing. Elements that participants considered important for socially functional community, for instance, trust between residents, tolerance to diversity, or rules, agreements and conventions that are planned together, were expected to be enhancers or enablers of functional space sharing. They considered communality requires management and social organizing. Routines of community of residents, that were related to shared spaces, for instance weekly activities, were considered beneficial for both communality and space sharing.

Participants believed that if residents feel being part of the community of residents, it advances the *experience of ownership* of the shared space, and further enhances *responsibility* and the use of shared spaces. Participants expected that if residents plan and agree together on the spaces or their use, furniture, rules, other agreements, etc. related to the spaces, and further, commit to the space, it creates a healthy foundation for the use of spaces.

*Problems in social interaction* were considered as a major source of complications in space sharing. Participants underlined that sharing in residential context is dependable on humans and how they operate together. Space sharing was considered to cause new issues or responsibilities to handle and questions to solve for the community of residents. Participants assumed both individuals and community causing problems in social interaction. It was seen as a constant challenge of community of residents tolerate the differences inside it, and this was considered to increase in the context of shared spaces. In addition, space sharing requires social skills from individuals; however, it was acknowledged that everyone does not have those skills. This causes a challenge for the community to stay balanced in conflicts, guard the atmosphere and socially organize.

Participants described that space sharing requires *rules and agreements*. Rules were also seen as complications. Residents might not know or care about the rules, or interpret them differently, or community of residents does not have an agreement on rules. Participants considered questions of the consequences of having rules as more difficult problems to solve. Control and supervision of rules was seen causing more tension between residents. Some participants experienced that sanctions could dilute trust between residents. Same applies to the reactions of the community members when rules are broken or even in a case of some mistakes with negative consequences. Some participants were worried that as an outcome of atmosphere of control residents become afraid of doing mistakes and relaxed attributes decrease.

Problems regarding *responsibility* was another major issue. Shared spaces were experienced to increase and complicate the questions of distributing responsibilities in housing companies. Participants considered that all users of the spaces have responsibilities in shared spaces; however, it was acknowledged that there is great variance in how seriously responsibilities are taken. Participants connected responsibility to mental *ownership*, namely experiencing that the space is theirs, not necessarily economically, but rather mentally. It was considered a problem in shared property in general, that people use it careless, because it is not experienced anyone’s own.

Absolutely the biggest problem […]is the question of ownership: Who owns them [shared spaces] mentally? Does everyone own them equally? Are they being owned so hard and with love that they are taken care of? And can the ownership be managed so that they are meaningful? (P1)

Participants saw space sharing as a possibility and *a challenge for the community of residents to develop*. The counteract between communality as the benefit and supporter of space sharing and problems in social interaction as a complicator was experienced to challenge the community for constant work for taking care of communality. The community was expected to mature and grow in trust, tolerance to diversity and equality. Space sharing causes reasons for the community of residents to plan, co-operate and agree together, for instance, on rules of using the spaces. Some participants saw possibility for individuals to learn social and co-operation skills, good behavior, responsibility, and sharing resources.

Participants expected increasing and advancing co-operation of residents as a major possibility of shared spaces. For instance, some participants considered that residents’ work for the maintenance of a shared space could work as a “buy-in”, namely, it would increase responsibility and the experience of mental ownership of the space. Also, social support and practical help between residents were expected to grow. Neighborhood communality was experienced something “that has been in the old times” (P8) and which allows “ringing neighbors doorbell and ask for sugar”. Participants considered there was a need to revive this communality and shared spaces were valued as an enabler of it.

#### Personal benefits and obstacles of space sharing

Although the community aspect in space sharing was emphasized in the interviews and participants were tending to treat the subject of space sharing more on a general rather than individual level, personal experiences and motivations and obstacles on individual level were nevertheless found. Considering individual benefits, participants *expected shared spaces to benefit their own mental and physical wellbeing*. This was associated with the activities participants expect they can do in the shared spaces (i.e., functions of shared spaces) and other possibilities they can offer, for instance possibility to meet others or getting out of one’s own home.

[in shared spaces] one can socialize with neighbors and other people, get out of own home. And then also this physical side as well, that one might want to go, or goes physically from home to some other place. And if that shared space happens to enable physical activities, it also adds to the pile of physical [wellbeing] (P5)

Participants experienced mentally refreshing to get out of their homes. Shared spaces were seen providing possibilities to do so, especially if the spaces would provide mental stimulus, for instance relaxing atmosphere, connection to nature, scenery, or some other visual stimulus. Participants explained that shared spaces could advance mental or social wellbeing by providing possibilities for social interaction, getting to know neighbors, and reducing loneliness. Getting to know neighbors was further connected to sense of personal safety or safety of family members because it was experienced as an additional network of social support. Physical wellbeing was associated with expected possibility to do physical activities like sports and exercise in the shared space. In addition, participants considered that any reasons to physically go to any shared space increases physical activity especially for older people. In addition, participants expected moderately saving personal costs because of space sharing, which has a connection to economic wellbeing.

*Personal obstacles in using shared spaces* related to other people or condition of the space. Some participant wanted to avoid other space users’ behavior that they were not comfortable with. Shyness was a factor for some participants to increase the threshold to use shared spaces. For instance, not knowing other people in a shared space, not knowing who would be there or threshold in starting a discussion were seen as obstacles for some participants. However, previously presented meeting during activities was considered fluent even by participants who described themselves shy. Shared spaces with functions or activities connected to them were expected to provide possibilities for accidental encounters with neighbors, which our participants considered positive. Shared spaces were considered to mitigate the experienced threshold or awkwardness of meeting other people in general.

The backyard barbeque place, it is quite nice that there are other people at the same time. […] I do not think myself as a very social person otherwise, so it is quite refreshing to get into these situations without myself organizing those anyhow. Afterwards I feel good about these encounters with others. (P11)

Further, spaces themselves might have attributes participants saw as an obstacle of using them. Unpleasant or unclean space reduced motivation to use them. Some participants based this on their experiences of the shared spaces they had seen: an image of a clubroom, that is partially used as a storage in a basement with old furniture that people have abandoned, was drawn from some participants experiences. Some participants associated risks for health and hygiene to shared spaces, due to the COVID-19 pandemic. Some considered shared spaces poorly maintained and on no-one’s responsibility.

### Residents’ perceptions of using intelligent technologies that advances the use of shared spaces (RQ2)

Related to residents’ perceptions of using intelligent technologies that advances the use of shared spaces three main themes were found: “Residents are open to new purposeful technologies regarding space sharing”; “Intelligent technologies can lower threshold to use a shared space by facilitating space use and activities” and “Intelligent technologies can help the community of residents to develop.”

**Fig. 4:**
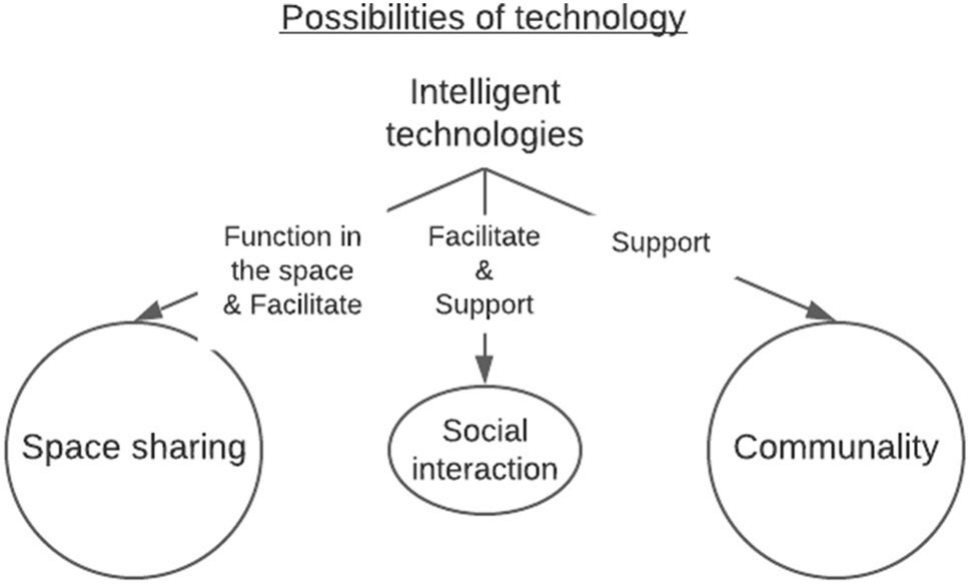
From the resident perspective intelligent technologies can support space sharing by having a purpose in the space, by facilitating space sharing, by facilitating and supporting social interaction and by supporting communality

Advancing the major enhancers of successful space sharing presented in Sect. 4.1, namely fluent use of the spaces and functional community, were seen as major needs and possibilities of intelligent technologies. Residents are open to intelligent technologies to be used for answering to the challenges of space sharing; however, they are skeptical of the capabilities of intelligent technologies to do so. From resident perspective, potential intelligent technologies could be categorized as following: technologies that have a distinct function in the space, technologies that facilitate space use and activities, and technologies that facilitate social interaction and support communality. Figure 4 illustrates the possibilities that participants expected intelligent technologies to have. In addition to residents’ perceptions to intelligent technologies, this section presents findings related to residents’ perceptions to technologies in general.

#### Residents are open to new purposeful technologies regarding space sharing

All participants were in general open to new technologies in living sphere and in shared spaces, assuming technologies are meaningful and answering the needs of space users and the purpose of the space. In general, technology was expected to bring some added value for the users. Participants mentioned for instance economic, ecological, and social value, benefits for wellbeing, safety, and making life easier as potential value that technologies in shared spaces could provide. However, participants experienced themselves somewhat unaware of possibilities of intelligent technologies and skeptical of their capabilities to meet the challenges caused by space sharing. For most, technology was considered as secondary in the context of space sharing.

Various possibilities for intelligent technologies were nevertheless found. For instance, smart guidance in activities or automatic transformation of adaptable multipurpose spaces were considered potential use possibilities for intelligent technologies. Another example is also related to saving costs and energy and further being environmentally sustainable: AI and automation were considered as a possibility to save energy in shared spaces by adjusting and controlling heating and lighting.

The most mentioned purpose of technologies related to space sharing overall was communication. Social media and other communication technologies were already highly used by participants for communication in the neighborhood, and they saw possibilities for those in the future. However, this does not implicate need for new technologies. Some participants experienced technology stress and a problem of having "too many apps". Some explicitly mentioned preferring existing platforms. In general technologies or purposes of technologies that participants related to shared spaces divided opinions. Technologies for entertainment were considered positive by participants, however, if it includes games, divided participants. Some were open and interested in for instance, virtual, augmented, and mixed reality or social robots and some were hesitant. In general, participants wanted to avoid technologies that would have low use rate or would have a short life span. For instance, some participants considered smart home automation as fast expiring technologies. Participants considered that technology could answer to the need for safety in shared spaces. They saw possibilities that technology could advance fire safety, security or work safety in for instance, handcraft space. However, especially surveillance technologies caused concerns related to privacy and mitigating trust in the community of residents. These expected use purposes of technologies related to space sharing continues similar thematic than residents’ expectations towards shared spaces presented in Sect. 4.1. Most participants were open to intelligent technologies to be used for same purposes, if applicable.

Also, intelligent technologies divided opinions. Some were positive towards automation with artificial intelligence, some felt fear towards it. However, it is worthwhile to notice that most participants had difficulties to think beyond the technologies they are currently aware of. Some participants found it also difficult to believe that intelligent technologies have capabilities to solve the issues of space sharing. Some intelligent technologies caused conflicts in participants individual thinking. For instance, in using artificial intelligence in living sphere, it was seen positive if it automates routine like activities and user does not have to think about or actively use the technology. However, it caused concerns if it is reliable, especially if it can make decisions related to safety.

Two *major concerns* that participants had towards technology in general, including intelligent technologies were privacy and data security. This concern was mostly connected to surveillance and tracking technologies or systems that users need to give great amount of information of themselves. Surveillance technologies and face detection were also experienced intrusive. Despite residents’ openness towards intelligent technologies, these concerns limit the user centered design of intelligent technologies in living sphere. In addition, problems in usability and accessibility concerned some participants. A major concern related to technologies in shared spaces indirectly was that it will become a target of vandalism or theft.

#### Intelligent technologies can lower threshold to use a shared space by facilitating space use and activities

A major theme of purposes, possibilities and user needs of technology in general related to space sharing was that technology can facilitate the use of shared spaces. This theme covers pragmatic outcomes of technology use, that make it easier for the residents to use the shared space. This was considered to advance the use of shared spaces by “reducing the unnecessary” work or tasks from the space users so they can be focusing on the actual reasons that motivate them to use the space, for instance activities or meeting other people. Participants were positive towards the idea of leaving unnecessary work for intelligent technologies to take care of. One previously discussed major need for shared spaces was them to be available and easy to access. Readily available reservation systems and electronic locks would advance the current state of many participants’ living environments; however, participants believed that technology could be used more broadly and with more intelligence to make spaces easier to go to.

Some participants acknowledged that technology could contribute to answering to the need for adaptable and multipurpose shared spaces. In those, intelligent technologies could be used for controlling the adaptiveness of the space based on particular use need. Some participants proposed that artificial intelligence, for instance, could estimate the space usage and control the space to adapt for the use automatically. In addition, automatically controlled visual elements or digital walls and floors in the space could adapt to different use needs or to make the space more pleasant and stimulative. Participants also considered that technology could assist in maintenance and utilities in shared space, for instance automate cleaning and communicate maintenance needs.

An instructive and informative role of technology was presented by multiple participants. Technology could provide information about the shared spaces and how to use the space. This was emphasized in the case of potential handcraft space or other space that has some tools or devices that users of the space can use. Artificial intelligence could be applied in making information about the space available and visible, for instance market the space and visualize statistics of space user, high usage peaks, etc.

#### Intelligent technologies can help the community of residents to develop

An important possibility that participants saw for intelligent technologies and technology in general was supporting communality or in other words, facilitating the development of community, which was seen important for successful space sharing. Many participants described existing technologies to advance communality, equality, and accessibility in their neighborhood, and said they expected that technology would serve that purpose in the future as well. However, considering intelligent technologies many participants were skeptical about the capability of it to support communality.

Participants emphasized the role of communication, and communication technologies in the development of community of residents. They experienced social media or communication technologies important in advancing communality in their neighborhoods. Simultaneously they experienced those as risks for the communality because of arguments, provocation, false information, and other misbehavior.

Participants described that technology could increase social interaction directly (i.e., technologies that provide possibility to communicate or otherwise interact with other people) or indirectly (i.e., as a reason to use the shared space, gather among the technology). As a part of increasing social interaction directly, some participants considered intelligent recommender systems that could match and recommend people spaces or activities. In addition, some participants proposed persuasive technologies for increasing the use of shared spaces and bringing people together.

However, participants saw also complications in this theme. They acknowledged the risk of people being overly focused on technology instead of other people. Some participants considered similar risk of overly high emphasis in technology in planning the spaces. Participants considered privacy important, and concerns related to it were connected to both living sphere and technologies. The importance of privacy was considered high in technologies related to community of residents. Same applied to data security.

### Use purposes and roles of intelligent technologies in advancing the use of shared spaces (RQ3)

Design implications (RQ3) are presented in two main themes that arose from the study: “Key purposes and concerns of intelligent technologies in shared spaces” and “Roles of intelligent technologies to advance use of shared residential spaces”.

After combining and analyzing the results of the interviews and the co-design workshops, key purposes, and roles of intelligent technologies, with some concerns towards intelligent technologies for advancing the use of shared spaces were found. These general perceptions were emphasized in the data of this study instead of detailed functionalities.

**Fig. 5:**
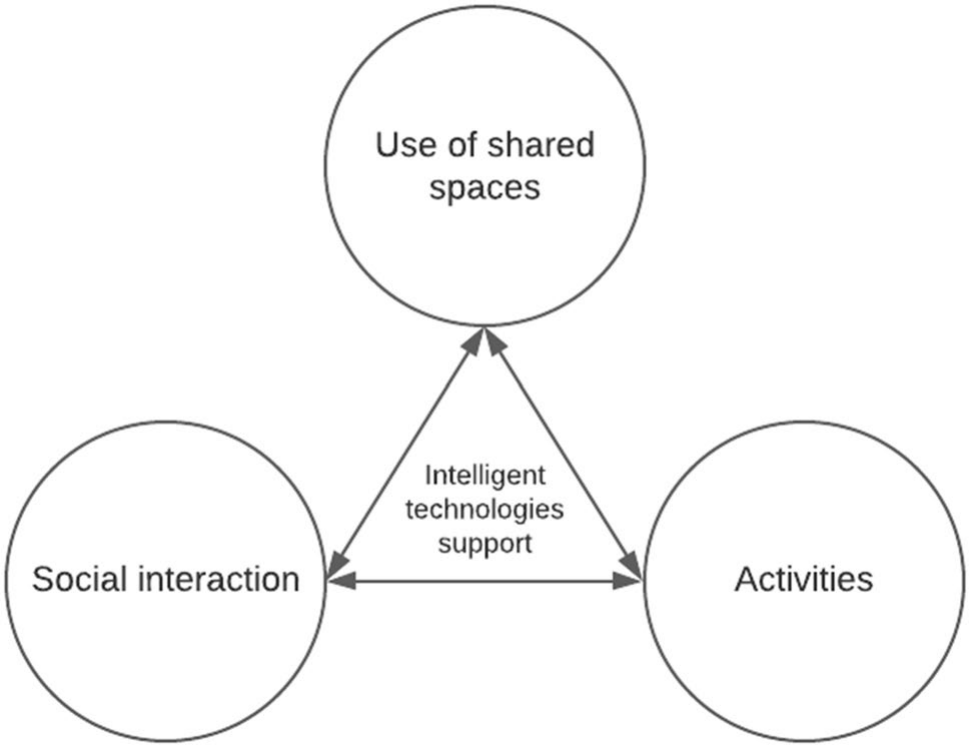
Intelligent technologies can connect and support the use of shared spaces, social interaction, and activities

Intelligent technologies were considered to have potential to enhance the factors that participants experienced to support space sharing and mitigate the expected problems. These supporting and challenging factors were presented in Sect. 4.1. Intelligent technologies can be used to support community of residents in social interaction that is needed in space sharing and to make using the shared spaces easier for the residents. Figure 5 describes how found use purposes of technologies link to the interconnections of the use of shared spaces, social interaction and activities that support each other.

#### Key purposes and concerns of intelligent technologies in shared spaces

The expert workshops produced ideas for use purposes for intelligent technologies based on findings of resident interviews, which were again evaluated in the light of interview findings. This resulted in several use purposes for intelligent technologies, that can be categorized as *intelligent technologies for organizing and facilitating*, *intelligent technologies for matching, recommendation and connecting*, and *supportive intelligent technologies for space and activities*.

Considering requirements and limiting factors of technology design, privacy and data security were high in residents’ concerns and those should be considered carefully. For instance, surveillance technologies and face recognition were experienced intrusive. Maintenance of the technologies concerned participants as an extra cost or an issue to be solved for the housing company. Usability needs are high in the context of space sharing in residential areas. Technologies that user does not need to pay attention to were favored.

*Intelligent technologies for organizing and facilitating.* Intelligent technologies can meet different needs of residents for organizing and facilitation. Technologies for organizing and facilitating was recognized to have several potential use purposes. First, it could organize and facilitate community and social interaction done or supported by artificial intelligence. For instance, conversational AI can activate and motivate to social interaction, moderate discussion, and promote positive interaction. AI can be an objective agent, gather people together and do the scheduling. Second, it could organize and facilitate space use practicalities. AI can organize and control space usage and maintenance, for instance, detect use rates and times and regulate the use by scheduling and possibly pricing fees of use. Third, it can organize events or activities, the needed resources and logistics, and invite people. This can advance efficiency, sustainability, and accessibility. In the case of adaptive multipurpose spaces intelligent technologies could be used to control and automate their adaptation. Fourth potential purpose was, that technologies could advance activities. Based on user and space usage data AI together with communication and social technologies can innovate, organize, and facilitate activities for residents. Finally, use purpose was that technologies could facilitate learning and guidance. AI and other intelligent technologies can teach, guide, or facilitate learning. It can for instance, instruct how to use a space or devices in it, guide in activities or social interaction, or facilitate learning by creating circumstances for learning, mediating knowhow or matching people with learning needs and knowhow.

*Intelligent technologies for matching, recommendation, and connecting* could serve three purposes. First, AI could detect common interests and values in the residential community. These data can be further used to develop for instance, activities in the spaces. Second, it could bring information visible, meaning that intelligent technologies can collect, analyze, visualize, and communicate information related to space sharing. Finally, it could match people, spaces, and activities, for instance, match people based on their user data and recommend common activities in a suitable shared space in the neighborhood. Suitable activities could be, for example, same aged children playing together, random coffee breaks or sports and exercise. It can match people with needed and existing knowhow or needed and offered help and assistance. Used data can include for instance activities that user does or desires to do in shared spaces, knowhow user possesses and is willing to share, space use times or demographic information. Recommending targeted activities, events, or shared resources for residents could be based on these data; however, this holds a risk of creating social bubbles.

*Supportive intelligent technologies for space and activities.* Three use purposes are categorized as supportive technologies. First, AI can advance safety and security by monitoring, controlling, and regulating for instance, work safety in handcraft space, health, and hygiene factors (e.g., safe distances for the pandemic), access control and identification. Risks with this, however, are that these kinds of technologies can be perceived intrusive and violating privacy. Second, all technologies that serve the functions of the space, e.g., utility technologies, entertainment, games, or tools in the space, are categorized here. They were considered potential in bringing people together around them. Third and finally, intelligent, persuasive, and gamified technologies can be used for motivating people for instance, to use shared spaces, to learn new, or to behave constructively and sustainably. They can for instance monitor and detect behavior and persuade towards chosen behavior by challenges and rewards.

The described potential use purposes of technologies are summarized in Table 1.

**Table 1:** Potential use purposes of intelligent technologies

Potential use purpose of intelligent technologies	Category
Organize and facilitate community and social interaction	Intelligent technologies for organizing and facilitating
Organize and facilitate space use practicalities	
Control adaptive multipurpose spaces	
Advance activities by innovating, organizing and facilitating	
Facilitate learning and guidance	
Detect shared interests and values	Intelligent technologies for matching, recommendation and connecting
Bring information visible	
Match people, spaces and activities	
Advance safety and security	Supportive intelligent technologies for space and activities
Motivate	
Serve the functions of the space	

#### Roles of intelligent technologies to advance use of shared residential spaces

Besides the more concrete level of use purposes, the experts in the workshops discussed the topic on a more abstract level. This discussion provided descriptions of roles, through which intelligent technologies can meet the needs and challenges that residents presented towards space sharing. The roles summarize the use purposes and provide goal and action oriented descriptions. Based on found use purposes and tentative roles found in the workshops, four roles of intelligent technologies are proposed: a community sheriff, a matchmaker, a facilitator, and a tutor. These roles aim at addressing the needs and challenges of space sharing that are presented in Sect. 4.1 with different forms of intelligent technology solutions.

**Community sheriff** Community sheriff works as an intelligent agent that can monitor residents’ behavior and activate them towards positive behavior and commonly agreed rules of the shared space. It observes and protects the communality regarding shared spaces through inspiring and motivating people to act in certain ways. Through gamification, give challenges and rewards for constructive behavior, responsibility and maintenance of the space, and sustainable choices. A sheriff can prevent exclusion and discrimination. It can activate people to use shared spaces and carry out various activities in them, or even innovate new activities.

The community sheriff could be present in the community through both a mobile app and public displays in different areas of the neighborhood. It could be an animated agent with speech interface and positive personality. It could learn about people’s behavior in a shared space and adjust its responses accordingly.

*Examples of use* Sensors in a shared space detects its cleanness after people have used it. Community sheriff gives reminders to the space users to clean up. AI follows trends in the spaces usage and maintenance and nudges the space users towards following the rules of the space in advance.

**Matchmaker** Matchmaker can match people, activities and spaces based on, for example, space usage times and habits, residents’ preferred activities, possessed knowhow and residents’ needs for and potential to help. Matchmaker recommends activities, events, spaces, and possibilities for helping or receiving help in various needs and help residents to share resources. Matchmaker promotes people with similar or complementary interests meeting each other, sharing knowhow, resources, and helping each other. This could be especially valuable for newcomers in the residential area.

A matchmaker can be implemented as an add-on application to social media service, sending push messages (allowed by the users) to present new possibilities to the residents when they are around their neighborhood. It could be embodied as a social robot in the shared space, with explicit privacy.

*Example of use* Matchmaker recognizes use preferences of the users of a repair and handcraft space. AI algorithm recognizes the user and asks to confirm the skills and knowhow they might possess. Based on possessed and needed knowhow, Matchmaker makes matches and proposes people who need and can provide help to each other, and they can be invited to the shared space. It can also suggest more specific activities based on user preferences.

**Facilitator** Facilitator lightens the residents’ work burden related to shared spaces by organizing space use practicalities. It can, for instance, control reservation systems, schedule the space usage and activities, and control space resourcing based on use rate and times to gain more efficient use rate. It can help facilitate space use by automating administrative tasks, control maintenance, logistics and resources. In adaptable multipurpose spaces AI can control and automate adaptivity.

Conversational AI can facilitate social interaction. It can take the role of an agent that brings people together for enabling them to do something together. It can organize activities or events and invite people there based on their preferences. Using IoT technologies, facilitator can bring information about shared spaces and make activities visible. It can provide information and advertise the events in shared spaces and recommend events and activities.

*Example of use* Facilitator organizes, optimizes, and automates the use of a rentable multipurpose space. Space has transforming elements, and everything that can be automated, is made ready for each activity that the space is reserved for, e.g., a volleyball practice or a knitting club. AI optimizes the times of use and times of low use rate are rented with lower prices. If some time slots remain empty even after this, system proposes them for individuals in the neighborhood that might be interested on a same hobby, for example floorball for fun.

**Tutor** AI tutor can teach and motivate residents to learn activities. Taught things can include the activities done in shared spaces, e.g., exercise, or handcraft or using the space or technologies themself. An intelligent technology tutor can also teach social and conversational skills. A tutor can also help people to teach each other by gathering pool of knowledge and recommend people to share their knowhow when it is needed.

Tutor could take a form of a social robot or chatbot. A conversational user interface that learns from the users’ communication with them is a prominent form of a tutor. A ML-based recommendation algorithm can help groups of people to learn together and from each other.

*Example of use* Tutor gathers a pool of knowledge in the residential area. It recognizes difficulties of use of the tools and functionalities of a shared space and generates instructions on specific activities, for example, how to grow chili in a shared garden.

## Discussion

This study investigated residents' perspective towards shared spaces and intelligent technologies advancing the use of the spaces, and based on the results, draws an image of communality and space sharing as supportive factors for one another, enabled and complicated by social interaction. This setting cuts across this study. Major possibilities for intelligent technologies advancing space sharing were seen in supporting the most remarkable enhancers of well-functioning space sharing, namely fluent use of spaces, activities in them and communality. The roles we present for intelligent technologies utilize these possibilities.

In the first study phase, resident participants expected (RQ1) shared spaces to be extensions of home that bring value regarding size or function. Space functions, activities in spaces and social interaction were tightly connected to participants’ expectations towards use of shared spaces. Activities were considered to catalyze meeting people, and further advance neighbors knowing each other and develop communality among residents. Participants expected feasibility from space use, for instance being easy to access and close to their homes. Unpleasant or unclean space and personal shyness were considered as obstacles of use. Positively for our topic, some participants who considered themselves shy, had had positive experiences in meeting people by coincident while doing some other activity in common areas of their residential building.

Although the context is different these pragmatic expectations towards shared spaces were, where applicable, parallel with Nugent’s (2012) findings of well-functioning shared spaces in student colleges. Especially proximity, presence of activities and experience of ownership were relevant findings in our interviews. Considering planning of shared spaces in Hiedanranta, this study proposes to plan shared spaces where residents can carry out activities. Following the needs of proximity and feasibility, multiple spaces distributed equally and close to residential buildings are preferred over one space in the middle of the area. Adaptability, pleasantness, and involving residents in their planning, organizing and adaptation are additional important factors to consider.

In addition to efficiency and personal wellbeing, enhancing communality was seen as a major benefit of having and using shared spaces in the neighborhood. On the other hand, communality was considered to support space sharing. Social interaction both enable and complicate space sharing among residents, which creates a challenge for the community of residents mature in communality, co-operation, social interaction, and taking responsibility.

In previous literature, increased social interaction was related to social workspaces (Chan and Zhang 2021; Seo-Zindy and Heeks 2017), placemaking process (Stupar et al. 2020), and various solutions in built environment (Kuo 1998; Curl et al. 2015; van den Berg et al. 2016). In our study, participants expected increased social interaction from using shared spaces.

Residents’ perceptions of using intelligent technologies (RQ2) that advances the use of shared spaces reflect the interconnection of space sharing, social interaction, and communality, that cut across the study. Possibilities for intelligent technologies were seen in the spaces, facilitating the use and sharing of spaces, supporting communality and facilitating and supporting social interaction. These results remained less detailed than expected. These intersections of place, people and technologies are familiar from the domain of urban informatics (Foth et al. 2011) and therefore, they are not surprising findings, nevertheless important in connecting this study to a larger framework.

This study investigated users’ perceptions in a context where intelligent technologies are new. Participants were in general open towards new technologies; however, they experienced themselves unaware of the possibilities of intelligent technologies. Although not a major finding, this was remarkable for the study, because it caused a change in study design. Originally, same participants were planned to participate both interviews and co-design workshops. For this early-stage user research, interviews worked well for exploring the use context and user needs. However, in the interviews participants experienced themselves not having enough knowledge to talk about intelligent technologies and the interviews turned out to provide descriptions of previous experiences and relatively general descriptions of expectations, especially considering the potential intelligent technology solutions (RQ2). In addition, considering the technology solutions, the answers remained general, even after probing with examples, mostly due to the participants’ relatively low level of awareness of intelligent technologies. Since the aim was to inquire into future directions of intelligent technologies, we decided to recruit people with more experience in artificial intelligence to the co-design workshops and use the rich findings on user’s expectations, needs and use context as stimulus material in the workshops for exploring issues of RQ3 on design implications. Using user experience (UX) and human–computer interaction (HCI) experts with experienced in artificial intelligence as participants also served the need for co-design workshop participants to understand the resident needs presented to them.

We considered having early adopters as participants in the interviews. It might have been more productive on findings; however, it would not have represented as well the variety of potential residents. In addition, we considered running workshops together with the non-expert participants. However, the expert workshops with UX and HCI expert who had some experience in AI was a benefit because these experts could take both the resident perspective and the potential of intelligent technologies into consideration. In addition, most of these participants also represented the group of residents of apartment buildings relatively similar than Hiedanranta. From the perspective of studying user experience, this arrangement is a step further from the user; however, the workshops managed to take the input from the interviews broadly into consideration. The further evaluation nevertheless is a matter of future research.

This study presents use purposes and roles of intelligent technologies. In their further evaluation and particularly in potential development of the technologies, it is important to notice the concerns of residents. Applying the roles of intelligent technologies can likely conflict with residents’ concerns about privacy, data security and intrusiveness, because they need data on residents to work properly. The question of who owns and manages the gathered data can have an impact on residents’ experiences (Mann et al. 2020) and should be evaluated further.

This study adds the resident perspective about shared spaces and technologies in them to the superblock discussion (Sjöblom et al. 2021; Rueda 2019) and already started unique resident participation to the planning process of Hiedanranta (Kuoppa et al. 2020; Laine et al. 2020; Sjöblom et al. 2021). This perspective brings the discussion on the intersections of place, people and technologies (Foth et al. 2011) closer to investigation of technology use in residential areas. Simultaneously, the findings contribute to the Human-Centered AI (HCAI) research, which is central in understanding the effects of AI to human everyday life (Schmidt et al. 2021).

### Future work

Future studies should validate and evaluate the proposed use purposes and roles of intelligent technologies in superblocks. The found roles require rich use of data gathered form user behavior. Empirical results of the roles can be investigated by implementing prototypes of the roles using state-of-the-art intelligent technologies and testing them in residential areas. In addition to the user experience, conflicts of privacy, and data security need to be investigated from the perspective of human-centered design. How willing are residents to allow gathering such data in their living sphere and will the benefits outweigh the concerns? For what reasons would residents allow data usage, and how would different modes of data ownership impact acceptance? Mann et al. (2020) describe citizens experiencing anxiety of control in the context of smart cities and as a respond they suggest technological sovereignty, self-governance of technology and data and emphasizing public and common interest rather than business interests. Further research of the privacy and data ownership questions in the context of residential space sharing is needed. For example, the algorithmic principles of recommendation systems require strong inspection and ethical consideration to avoid creating social bubbles, amplifying stereotypes and discriminative patterns. In addition, this study calls for research and design on how technologies in general could support residents in taking responsibility and ownership of shared living spaces. Finally, in the light of the pandemic era, the concepts for safe encounters with the possibilities to avoid close physical contacts in shared spaces should be explored.

## Conclusion

Space sharing is interconnected with communality: they support each other, and they both are enabled and complicated by social interaction. The use of shared spaces is motivated and further supported by functions and activities in spaces, as well as by the possibility to meet other people in the neighborhood.

Intelligent technology solutions may advance space sharing by facilitating fluent use of spaces, activities in them, social interaction and communality. AI solutions have potential to execute these possibilities through the roles found in this study: community sheriff, matchmaker, facilitator, and tutor. These roles could be implemented through different intelligent technologies to act according to the roles: as an objective observer that gently pushes people towards positive behavior; as an agent that matches, recommends and connects people, places and activities; as a facilitator that organizes and removes obstacles and extra work, and a tutor that teaches and motivates residents in their shared activities.

In Hiedanranta and other future superblocks, intelligent technology solutions can serve a variety of use purposes and have the presented roles; however, they require validation through empirical evaluation. From the resident perspective, these kinds of solutions can advance sustainability through promoting the use of shared spaces. Sustainable outcomes can be achieved through resource efficiency, and by motivating residents towards sustainable actions. Outcomes supporting residents’ wellbeing result from increased activity, social interaction, and communality. However, the mentioned benefits manifest only if spaces are used, and technology-related concerns such as privacy are taken care of. Continuous involvement of residents in the processes of planning, developing, and maintaining the spaces, as well as in the design of technologies is encouraged. This will enable the human-centeredness of future AI solutions for the benefit of people and sustainability of the urban living spaces.

## Data Availability

Fully anonymized data are available from the corresponding author with a reasonable request.
